# Retrospective radiographic myelogram measurements and long-term outcomes in horses undergoing cervical interbody fusion surgery: 22 cases

**DOI:** 10.1371/journal.pone.0323083

**Published:** 2025-05-07

**Authors:** Devon England, Lauren Newsom, Constance White, Erica McKenzie

**Affiliations:** 1 Department of Clinical Sciences, Carlson College of Veterinary Medicine, Oregon State University, Corvallis, Oregon, United States of America; 2 Department of Biomedical Sciences, Carlson College of Veterinary Medicine, Oregon State University, Corvallis, Oregon, United States of America; Long Island University - CW Post Campus: Long Island University, UNITED STATES OF AMERICA

## Abstract

Site selection for cervical stabilization surgery in horses with spinal ataxia frequently relies on measurements derived from radiographic myelography. A variety of measurement criteria exist and can provide conflicting results. The main objectives of this study were to assess the correlation between two commonly used myelographic measures, dorsal contrast column reduction (DCCR) and dural diameter reduction (DDR), and their association with previously selected operative sites in a population of horses operated at a tertiary clinic. Secondary objectives were to determine if articular process joint (APJ) atrophy occurred in a subset of operated horses with radiographic follow-up, and to describe complications of cervical stabilization surgery and long term outcomes. The study was primarily cross-sectional using previously recorded medical information and images from horses operated between 2008 and 2022: three masked raters assessed previously acquired pre-operative myelograms obtained in neutral, flexed and extended neck positions from horses that had subsequently undergone stabilization surgery consisting of cervical interbody fusion via a Kerf-cut cylinder technique at one or two sites. A veterinary radiologist evaluated changes in APJ in radiographs obtained in a subset of horses re-evaluated >18 months after surgery. DCCR was unremarkable at nearly all articulations in all horses, while DDR met reduction criteria at over 50% of articulations in flexed position. Neither DCCR nor DDR distinguished operated from non-operated sites at most intervertebral junctions, except at the C6-7 articulation in neutral and extended position. The two measures were also poorly correlated at most sites and in most positions. Surgical complications included a high incidence of laryngeal hemiplegia. Comparison of operated to non-operated sites within individuals radiographed years later showed consistent, mildly reduced APJ opacity at most operated sites without a consistent decrease in APJ height or area ratios. Our results suggest that DCCR and DDR measures did not reliably predict surgical site selection in this surgical cohort except at C6-7, and that the two measures yielded conflicting diagnostic classification at many sites and positions. Complication rates from stabilization surgery were high; and predictable reduction in APJ height or area after surgery was not demonstrated by radiography in this study.

## Introduction

Cervical vertebral myelopathy (CVM) is the most common non-infectious cause of spinal ataxia in horses, with ataxia arising from compression of the spinal cord at one or multiple locations within the cervical vertebral canal [1]. Preliminary diagnosis involves assessment of plain radiographs for malformation, malalignment, and acquired abnormalities of the cervical bones and joints, commonly accompanied by calculations of intravertebral sagittal ratios [2–4]. Definitive evaluation of spinal cord compression premortem is currently most often established from radiographic myelography [1,3,4]. Myelogram images are typically obtained with the neck placed sequentially into neutral, flexed, and extended positions to permit assessment of dynamic (variable with head position) and static (persistent) narrowing of the spinal canal. Compression is reflected by narrowing of the subarachnoid space on myelography, the severity of which can be determined via several different measurement methods [1,3,5].

Most recently, measurement of dorsal column contrast reduction (DCCR) or of total dural diameter reduction (DDR) on lateral-lateral radiographs are recommended in assessment of radiographic myelograms [3,4,6–10]. These methods are replacing or supplementing a prior practice of determining reduction in both dorsal and ventral contrast columns concurrently at a specific site [1,4,5,10–13]. For cervical vertebral articulations C2-3 through to C6-7, values of ≥50% and ≥20% for DCCR and DDR, respectively, have traditionally been considered abnormal [4,6,7]. Values of ≥60% or ≥30%, for DCCR and DDR, respectively, are now recommended for evaluation of the cervicothoracic junction (C7-T1) to avoid false positive assessments [10].

Surgical treatment of CVM commonly involves stabilization of selected cervical articulations via intervertebral implantation of a Kerf-cut cylinder or, less commonly, through plating or polyaxial pedicle screw and rod implantation techniques [9,12,14,15]. In horses with dynamic compression, typically identified at the more cranial articulations (C3-4 and C4-5), improvement in clinical signs is attributed to stabilization of dynamic compression, potentially combined with local remodeling of bone and soft tissues [5,16]. In horses with static compression, which is more commonly identified at caudal cervical vertebral articulations (C5-6 through C7-T1), it has been reported that regression of bony arthritic changes after 12 months of immobilization likely reduces neurologic signs attributable to compression [9], however, imaging assessment of bony changes in response to prolonged stabilization have been rarely reported. Complications of surgical stabilization are common and range from relatively benign to severe and fatal [2,5,11,15,17].

Since surgical stabilization is associated with risk, expense, and prolonged recovery times, and because multiple compressive sites commonly occur within an individual, it is important to reliably determine the most likely site, or sites, of compression [1,5,18]. Multiple clinicians, often with differing perspectives and expertise, are often involved in decision-making on an individual patient. This can include residents, internists, radiologists, neurologists, and surgeons, who may emphasize different measurement techniques or criteria when assessing the patient and the images. Furthermore, repeatability of methods can be variable, and there can be discordance between different observers and in the conclusions generated from different methodologies [18–20]. Since DCCR and DDR are currently the most frequently utilized measures for detecting potential spinal cord compression on radiographic myelograms in horses, our primary objectives were to discover if currently published diagnostic criteria for these measures of compression corresponded with the site(s) previously selected for stabilization in this surgical cohort; to determine how closely these measures correlate with each other across different neck positions, and their concordance in classifying intervertebral sites as potentially abnormal. We also evaluated complications specifically related to cervical interbody fusion, and long term radiographic changes and outcomes in operated horses available for follow-up.

## Materials and methods

### Horses

The medical records database of Oregon State University Lois Bates Acheson Veterinary Teaching Hospital was electronically searched to identify all horses that underwent surgical cervical stabilization at the institution between 2008 and 2022. Surgical site(s) selection was originally determined after assessment by the radiologist and internist on clinical service at the time each case presented, collaborating with the operating surgeon and using the same radiographic images that were evaluated in this study. All surgeries were performed by the same board-certified equine surgeon (Diplomate American College of Veterinary Surgeons; DACVS) specializing in equine surgical cervical stabilization. All horses in this study had surgical correction performed via intervertebral implantation of a Kerf-cut cylinder at one or two cervico-cervical intervertebral articulations in a single surgical event [9]. Relevant data from operated horses, including signalment, bodyweight, neurologic ataxia grades before surgery (modified Mayhew Scale, [21]), and complications from surgery were extracted from medical records. Owners of all horses in which surgery had been performed a minimum of 18 months or more prior to the study were subsequently contacted to determine if horses were alive and available to return for evaluation and repeat cervical radiographs for the purposes of this study, or if post-operative images were available from other clinics. This study was approved by the Institutional Animal Care and Use Committee (IACUC-2021–0149) and owner consent was obtained for assessment of returning post-operative horses with owners signing an IACUC-approved consent form describing the study prior to inclusion of their animal. No animals were sacrificed for the purposes of this study. Assessment by the committee at Oregon State University that oversees research on human subjects determined that the project did not meet the definition of “human subject” under the regulations set forth by the Department of Health and Human Services 45 CFR 46.

### Myelogram evaluation

Flexed, neutral and extended neck position myelogram images obtained from all horses prior to surgery were anonymized by removing identifying names and data from images prior to re-uploading the films, assigning each horse with a randomized number label. Images were stored and measurements calculated using electronic calipers in NOVARAD (NOVARAD Enterprise Healthcare Solutions, American Fork, UT). All images were independently evaluated by three observers masked to each horse’s identity and each other’s results: a board-certified radiologist (LN – Diplomate American College of Veterinary Radiologists; DACVR), an internist (EM – Diplomate American College of Veterinary Internal Medicine DACVIM) and a third-year resident in an ACVIM-approved large animal internal medicine program (DE). Each observer made independent measurements at articulations C2-3 through C6-7 in the neutral and flexed neck position images, and from C4-5 through C6-7 in extended neck position images. The C7-T1 site was not assessed in this study.

Measurements of DCCR and DDR were made concurrently at each intervertebral site in each image using previously published methods, and values were reported as percentage reduction relative to measurement at the mid-vertebral body cranial to the articulation [6,7,10]. Calculation of DCCR was performed by measuring dorsal contrast column width at the mid-vertebral body (mid) and at the narrowest point over the adjacent caudal intervertebral space (IVS) for the equation [(DCC mid - DCC IVS)/DCC mid] x 100 [6,7,10]. Positive values for DCCR indicate narrowing of the dorsal column at the IVS, whilst negative values for DCCR using this equation indicate widening of the dorsal contrast column at that location. Calculation of DDR on myelogram images was performed by measuring the total height of the subarachnoid space at the mid-vertebral body (mid) and at the narrowest point over the adjacent caudal intervertebral space (IVS) for the equation [(DD mid - DD IVS)/ DD mid] x 100 [6,7,10]; positive DDR values indicate narrowing of the DD at the IVS relative to DD at the mid-vertebral body.

Mean DCCR and DDR value for each site was calculated from the three observers’ submitted measurements. A mean DCCR value ≥50% at any site in any neck position (and where 2 or 3 of the individual observer values were also ≥50%) was classified as abnormal for the purposes of this study [3,6,10]. A mean DDR value ≥20% at any site in any neck position (and where 2 or 3 of the individual reviewer values were also ≥20%) was classified as abnormal for the purposes of this study [3,6].

### Post-operative radiographs

**Fig 1:**
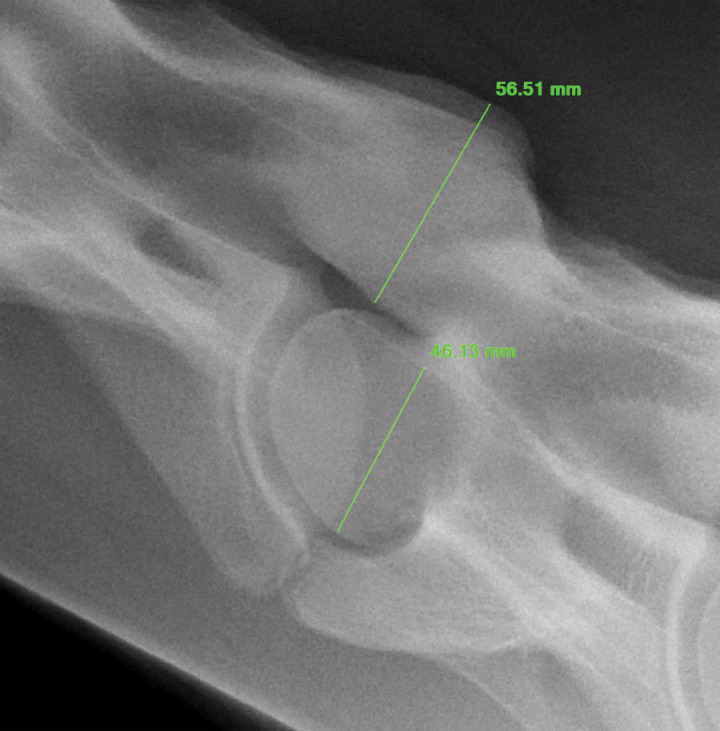
Measurement of articular process joint height and vertebral body height to create APJ height ratio.

**Fig 2:**
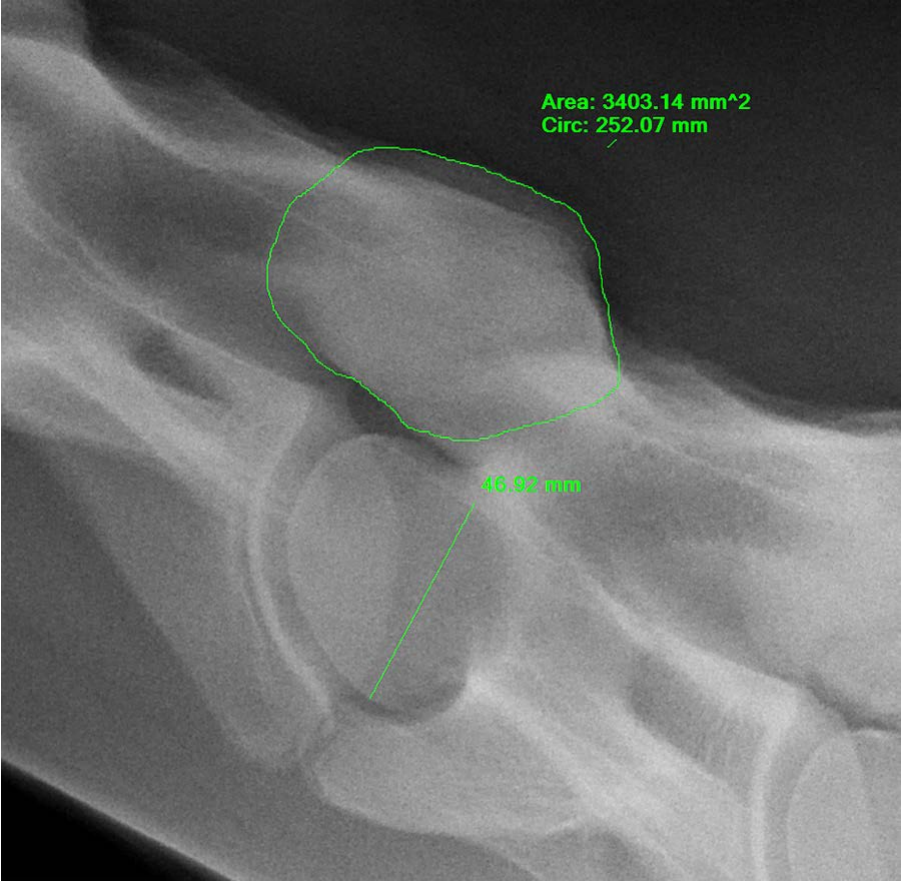
Measurement of articular process joint area and vertebral body height to create APJ area ratio.

Owners of horses operated more than 18 months prior were contacted to determine if their horses were available to return for neurologic evaluation and standing cervical radiographs, or if images obtained from horses more than 18 months post-operatively were available from other clinics. Two observers (EM, DE) performed standard ambulatory neurologic evaluation with ataxia grading (modified Mayhew scale) prior to sedation (xylazine 0.4 mg/kg IV or detomidine 0.01 mg/kg IV) to facilitate obtaining standing cervical radiographs. Articular process joint area, shape, and opacity were evaluated by the radiologist (LN - DACVR), who was masked to the identity and outcome of the horse. A ratio of APJ height and APJ area over the height of the cranial aspect of the caudal vertebral body was calculated by the radiologist on pre-surgical plain cervical radiographs and post-operative radiographs to determine changes in APJ size over time, while adjusting for differences in magnification between films obtained at different time points for the same horse (Figs 1 and 2). Thickness of APJs could not be assessed. Subjective assessment of opacity at operated compared to adjacent non-operated sites within available post-operative radiographs was also recorded.

### Statistical analysis

Statistical analysis was performed using SAS/STAT 15.2 software, and Stata/IC 16.1. Normality of data was assessed with the Shapiro-Wilk test. For normally distributed data, between-group means were statistically compared using independent t-test; within-group differences were statistically compared using paired t-test. When data did not meet normality assumptions, between-group ranks were statistically compared using Wilcoxon rank-sum test (Mann-Whitney U); within-group ranks were statistically compared using Wilcoxon signed rank tests. Statistical significance was set at p ≤ 0.05, with Bonferroni correction for multiple comparisons applied where appropriate. Correlation between DDR and DCCR measurements were performed using Pearson’s method, with confidence intervals calculated [22]; in cases where data did not meet assumptions of normality, Spearman’s correlation coefficient was also calculated. Agreement of raters for sites assigned as positive (‘abnormal’) using previously established criteria (DCCR ≥50%, DDR ≥20%) [6] was assessed using percentage agreement and Gwet’s AC1 [24]; in addition, raw ratings were assessed for agreement using Shrout & Fleiss (ICC 2,1 two-way random effects) intraclass correlation coefficients for absolute agreement. Interobserver agreement statistics were generated using Stata/IC 16.1 [23]. Intraclass correlation coefficients for raw radiographic scores between observers were calculated using Stata/IC 16.1 using two-way random effects (ICC(2,1)) for absolute agreement. Non-parametric receiver operator curves with associated indices were generated using Stata/IC16.1. Confidence intervals for sensitivity and specificity were generated using Stata/IC16.1; for the purposes of this analysis, the mean measurement of the raters at each site was used to classify sites as “positive” (potentially compressed) or “negative” [24].

## Results

### Horses and surgical sites

Twenty-two horses in which cervical stabilization surgery was performed between 2008 and 2022 were identified (Table 1). Warmblood horses (n = 13) predominated with the remaining breeds comprising Quarter Horses (n = 2), Tennessee Walking Horses (n = 2), Arabian (n = 1), Foxtrotter (n = 1), Irish Sport Horse (n = 1), Shire-Thoroughbred cross (n = 1) and Paint (n = 1). The majority were geldings (n = 18), and the remainder were mares (n = 4). Age at pre-surgical evaluation was 7.7 ± 3.9 years (range 2–17 years) and bodyweight was 564 ± 63 kg (range 430–642 kg). Neurologic evaluation during initial diagnosis before surgery included organized assessment of cranial nerve function, and routine gait evaluation maneuvers for the detection of weakness and proprioceptive deficits. Maneuvers included assessment of gait during backing, walking in patterns on flat ground, navigating an obstacle (a curb), and during application of an intermittent tail pull and head elevation. Reported grades of pelvic limb ataxia (modified Mayhew scale) recorded before surgery were 1 out of 5 (n = 1), 2/5 (n = 14) and 3/5 (n = 7) accompanied by grade 1 (n = 16) or grade 2 (n = 6) forelimb ataxia (Table 1). Pre-operative diagnostic imaging procedures performed on all included horses included plain cervical radiographs, and radiographic myelography (Table 1). Two horses also had pre-operative CT myelography images available (Table 1). Other relevant diagnostic procedures reported in the records included cytological evaluation of cerebrospinal fluid (CSF, n = 18) and testing for equine protozoal encephalomyelitis (CSF: n = 16; serum: n = 2) with no reported abnormal results except for one horse (horse #38) with a CSF SAG 2,3,4 titer of 1:20 and serum:CSF ratio of 100 against *Sarcocystis neurona*.

**Table 1:** Clinical and initial imaging findings on operated horses before surgery.

Horse ID & operative sites	Sex	Breed	Age at surgery	Clinical signs	Ataxia grade forelimbs	Ataxia grade pelvic limbs	Specific neurologic examination findings	Relevant findings by original radiologists
#4 C4-5	G	Tennessee Walking Horse	12	Stumbling, falling since purchase 2 years prior.	1	2	Dysmetric & toe dragging all limbs. Circumduction, pivoting behind. Reduced panniculus.	NSR; ‘mild narrowing’ of dorsal contrast column at C4-5 (flexed).
#6 C5-6 C6-7	G	Paint	5	Progressive resistance to backing over years.	1	1	Dragging all feet when backing, proprioceptive deficits,	SR of 0.45 at C4-5 & C5-6, DCCR 50% at C6-7 (extended).
#8 C4-5 C6-7	G	Oldenburg	13	Progressive tripping over 6 months.	1	2	Reduced proprioception all limbs, weaker left tail pull.	NSR; facet remodeling and mild steps at C5-6 & C6-7, DCCR 45% C4-5 (flexed).
#10 C5-6 C6-7	G	Arab	11	Tripping, ‘locking of neck’ over years, falling more recently. Traumatic neck injury at 5 months of age.	2	3	Abnormal stance, all toes scuffed and clipping when walked. Hesitant backing, circumduction on circling.	Facet remodeling at C5-6 & 6–7; SR < 0.5 at C6-7 with DCCR 50% (extended),
#11 C3-4 C4-5	G	Shire-Thoroughbred cross	8	Ataxia noted at pre-purchase examination.	1	2	Abnormal limb placement when standing, and pivoting behind. Right hind abnormalities more prominent.	Subjectively, ‘slight dorsoventral compression’ at C4-5.
#12 C3-4 C4-5	G	Warmblood	5	Tripping, falling over 1 month.	1	2	Drags right hind when backing, uncoordinated behind on circling, abnormal limb placement.	NSR; ≥ 50% DCCR at C3-4, C4-5 & C5-6 (flexed) & C6-7 (extended); mild step C3-4.
#19 C3-4 C4-5	F	Hanoverian	2	Progression of hind end weakness, stumbling and abnormal stance over 1 year.	2	3	Occasionally clips hind toes, hypermetric all limbs, abnormal limb placement, drags all feet when backing, circumducts on circling, weak tail pull bilaterally.	NSR; small step C6-7. Small deviation of ventral subarachnoid space at caudal end C5 & C6.
#22 C5-6 C6-7	G	Trakehner	8	Unusual foot placement, decreased jumping capacity (Grand Prix) over 6 months, 1 episode of ataxia.	1	2	Hesitant backing and dragging all four feet, misplaces feet over curb, pivots and circumducts behind on circling, abnormal placement, reduced panniculus.	NSR; 50% DCCR at C6-7 (neutral). Moderate dynamic instability at C4-5, C5-6, C6-7 (steps in flexion).
#23 C5-6	F	Westphalian	8	Acute onset neck pain, dragging all toes, difficulty lying down during training.	2	3	Abnormal positions, crouching behind and drags front feet when backing, interference over curb and during circling.	SR < 0.5 at C4-5, C5-6 & C6-7. Mild subluxation C6-7. DCCR 50% at C5-6 (flexed).
#24 C3-4 C4-5	F	Trakehner	8	Stumbling, cross cantering, bunny hopping and abnormal foot placement over 1 year, fell on lunge.	1	2	Slight hypermetria in front, abnormal foot placement behind. Reluctant backing intermittent forelimb dragging. Interference over curb, reduced strength on tail pull.	NSR; DCCR 45% and DDR 30% at C4-5 (flexed).
#27 C4-5	G	Warmblood	8	Abnormal R hind gait for 6 months since importation.	1	2	No comments in record beyond grade.	NSR; DCCR 50% at C4-5 (flexed) with subluxation.
#29 C6-7	F	Hanoverian	8	Gait abnormalities, cross firing, R hind limb drag at canter for 6 months since purchase.	1	2	Dragging hind toes, clipping the curb, reluctant to back, pivoting, circumduction.	SR < 0.5 at C3-4 & C5-6. DCCR 50% at C4-5 (flexed, neutral) and C6-7 (extended, with DDR 35%).
#30 C4-5 C5-6	G	Foxtrotter	17	Acute onset limb splaying and crossing, stumbling over 3 weeks.	1	2	Interfering on curb, dragging forelimbs when backing, mild circumduction.	NSR; > 50% DCCR at C3-4, C4-5 & C5-6 (flexed).
#31 C4-5 C6-7	G	Irish Sport Horse	9	Rotational fall jumping at 1.1 m show, then ataxia. Presented for surgery 5 weeks later based on external CT myelogram results from 2 days after fall.	1	2	No comment at pre-surgical assessment. Evaluation 2 days after injury: Abnormal stance all limbs, hypermetric forelimbs, hypometric behind, dragging during backing, ataxic and pacing with head elevated, grade 3/5 all limbs.	Loss of ventral and dorsal columns at C4-5 (flexed) on radiographic myelogram; equivocal dorsoventral cord narrowing & loss of ventral subarachnoid space at C4-5 & C6-7 on CT myelogram.
#32 C6-7	G	Mecklenberger	14	Progressive ataxia over 1 year, knuckling.	1	2	Dragging hind toes on backing, pivoting on forelimbs, circumduction behind, hypermetric in front downhill and when head elevated, abnormal foot placement.	NSR; DCCR 50% at C5-6 (extended) & C6-7 (extended, neutral), improves at both sites with flexion.
#35 C4-5	G	Warmblood	6	Stumbling, foot dragging that developed over 2 months.	2	2	Interfering on curb, dragging forelimbs when backing, mild circumduction behind, pivoting on forelimbs.	NSR; mild dynamic step defects of C3-C4, C4-C5, and C5-C6; no extradural cord compression.
#37 C6-7	G	Quarter Horse	9	Knuckling behind and buckling in front, multi-limb interference at walk over 3 years.	2	3	Uncoordinated over curb, knuckling behind, dragging all feet, hesitant downhill, inconsistent stride length, dysmetric when head elevated.	NSR; DCCR 56% at C6-7 (extended).
#38 C4-5	G	Quarter Horse	5	Shifting weight behind, parking out hind limbs, reluctant to work, fell once in the 10 months since purchase.	1	2	Abnormal stance, toe dragging when backing, hypermetria during downhill and over curb.	NSR; DCCR 33% at C4-5 (flexed), where an internist reported DDR > 20%.
#41 C5-6	G	Dutch warmblood	4	Knuckling, falling 2 months after purchase.	1	2	Mild hypermetria and floating in front limbs, exacerbated by head elevation, clipping the curb, circumduction, backing is resistant and uncoordinated, variable stance, weak tail pull.	NSR; Bilateral absence of transverse processes of C6. DCCR 47% and DDR 40% at C5-6 (flexed); DDR 26% at C4-5 (flexed).
#43 C4-5	G	Tennessee Walking Horse	5	Abnormal gait, including bunny hopping with progressive signs over 2 years since purchase, possible neck injury as a foal.	2	3	Truncal sway, varying foot placement and stride length. Hypermetric with head elevation. Backs slowly, noticeable circumduction with some pivoting and dragging.	NSR; DCCR >50% at C2-3 & C4-5 (flexed); column at both sites only 1 mm high.
#44 C2-3	G	Oldenburg	3	Slight left head tilt when ridden, suspected C2-3 OCD lesion noted on pre-purchase radiographs 1 month prior.	1	3	Tightrope walking in hind limbs, abnormal positioning when standing, intermittent tripping on curb.	On radiographic myelogram DCCR normal all sites, DDR 25–30% at C3-4 and C4-5 (flexed). On CT myelogram OCD of C2-3 right articular processes with mild dural impingement (grade 1).
#67 C6-7	G	Warmblood	2	Weakness, stumbling since 9 months of age.	1	3	Truncal sway, hypermetria of forelimbs, erratic limb placement, toe drag, pacing at trot. Circumduction and dragging hind toes on circling.	NSR; dorsal and ventral ‘column narrowing’ at C6-7 in neutral and extension, alleviated in flexion.

NSR = normal sagittal ratios.

SR = Sagittal ratio.

DCCR = Dorsal contrast column reduction (>50% considered abnormal for the purposes of this study).

DDR = Dural diameter reduction (>20% considered abnormal for the purposes of this study).

**Fig 3:**
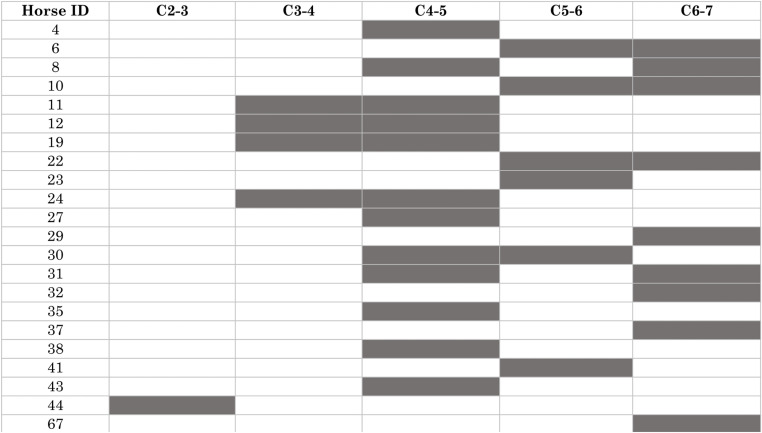
Operated sites in 22 horses undergoing surgical cervical stabilization. Grayed box = operative site, white box = non-operative site.

Twelve horses were operated at a single site and ten horses at two sites (Fig 3). The most commonly operated site was C4-5 (n = 12) followed by C6-7 (n = 9), C5-6 (n = 6), C3-4 (n = 4), and C2-3 (n = 1). Combinations in horses operated at two sites were C3-4 with C4-5 (n = 4); C4-5 with either C5-6 (n = 1) or C6-7 (n = 2); and C5-6 with C6-7 (n = 3).

### Radiographic myelogram measurements

#### Intervertebral junction C2-3.

**Fig 4:**
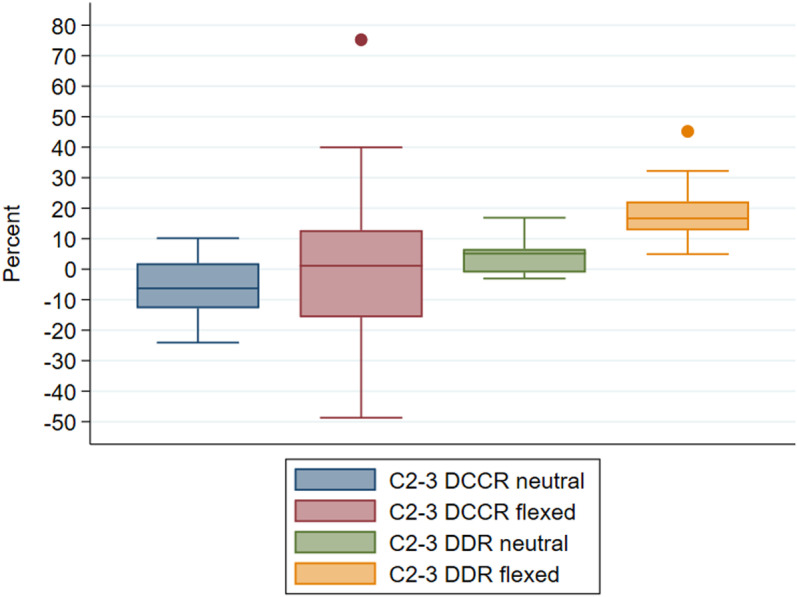
Mean observer DCCR (%) and DDR (%) at C2-3 in neutral (n = 22) and flexed (n = 18) neck positions. Horizontal line reflects the median.

DCCR and DDR measurements in neutral position were available for all horses at this articulation; measurements in flexion were available for 18 of the horses (S1 Dataset, Fig 4). All horses had unremarkable (<50%) mean DCCR values at C2-3 in neutral and flexed views, except for a single individual (DCCR 75.2%, DDR 45.2%) that was not operated at this site. Mean DDR at C2-3 exceeded 20% in 8 individuals (range 20.7%-45.2%) in flexed, but not neutral, views. A single horse was operated at C2-3, in a single site procedure, because there were changes consistent with osteochondrosis dissecans of the C2-3 right articular processes with mild dural but no cord impingement identified at this site on computed tomography performed after radiographic myelography. Mean DCCR (-9.7%) and DDR (16.5%) at C2-3 were unremarkable on radiographic myelography in this horse, though DDR was very high at C3-4 (DDR 34.4%, DCCR 13.1%) and C4-5 (DDR 36.8%, DCCR 34.3%) in this individual.

DCCR measures in all horses were not statistically significantly altered by flexion at this site, as compared to neutral (absolute mean difference 7.3%, paired t-test p = 0.1715); DDR was statistically significantly increased (reduced dural diameter) in flexion as compared to neutral (absolute mean difference 14.2%, paired t-test p = 0.0000), though this variable maintained a mean value slightly below 20%.

#### Intervertebral junction C3-4.

**Fig 5:**
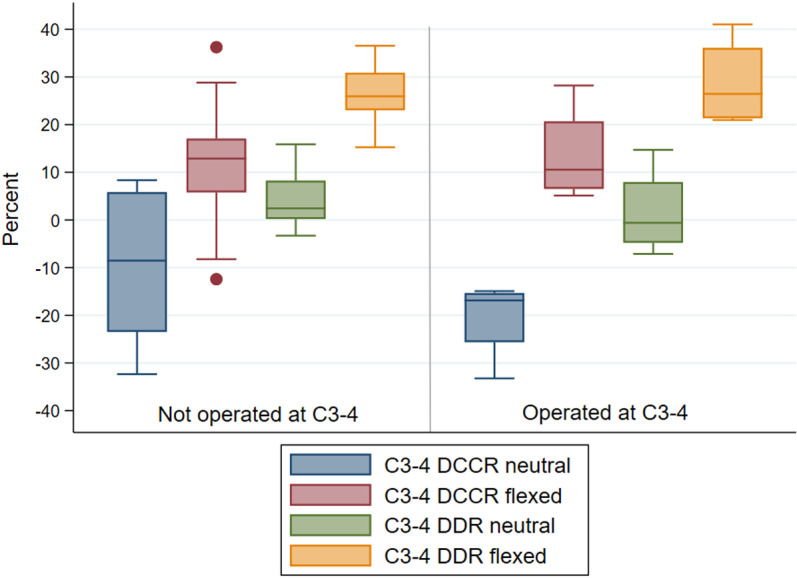
Mean observer DCCR (%) and DDR (%) at C3-4 in neutral and flexed neck positions in horses that were not operated (n = 18) versus were operated (n = 4) at this site. Horizontal line reflects the median.

Measurements were available for 22 horses at this articulation (S2 Dataset). No myelographic measures (Fig 5) were statistically significantly different between horses operated at C3-4 (n = 4) and those that were not operated at the site (n = 18, S2 Dataset). At this site, all horses had DCCR values <50% in both neutral and flexed positions, including the four horses operated at this site. All horses also had mean DDR values <20% in neutral position. However, mean DDR values at C3-4 in flexed position exceeded 20% in 18 of 22 horses (range 21.0%-41.0%; however, in one of these horses, only one of the three observers measured DDR as ≥ 20%; Observer 1: 17.9%; Observer 2: 19.0%; Observer 3: 26.0%).

Both mean DCCR and DDR values statistically significantly increased (reduced in diameter) in flexion as compared to neutral position for all horses (DCCR flexed versus neutral absolute mean difference 25.2%, paired t-test p = 0.0000; DDR flexed versus neutral absolute mean difference 23.6%, paired t-test p = 0.0000); mean DCCR did not exceed 50% in flexion at C3-4, though mean DDR exceeded 20% in this position.

#### Intervertebral junction C4-5.

**Fig 6:**
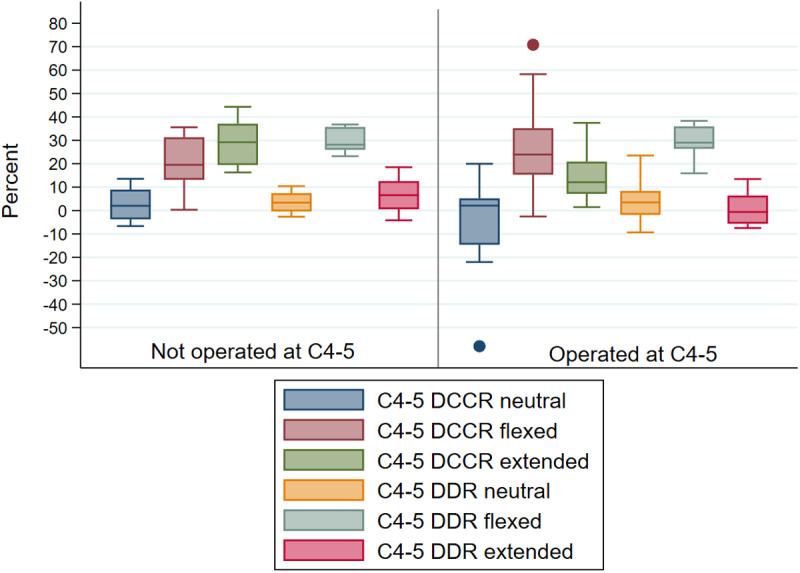
Mean observer DCCR (%) and DDR (%) at C4-5 in neutral, flexed and extended neck positions in horses that were not operated (n = 10) versus operated (n = 12) at this site. Horizontal line reflects the median.

Measurements were available for 22 horses at this site (S3 Dataset) which was the most frequently operated site. Apart from DCCR values in extension, myelographic measures (Fig 6, S3 Dataset) were not statistically significantly different between horses operated at C4-5 (n = 12) and horses that were not operated at the site (n = 10). DCCR in extension was statistically significantly smaller (greater diameter) in horses operated at C4-5 as compared to horses not operated at that site (absolute mean difference 14.4%, independent t-test p = 0. 003).

C4-5 DCCR mean values did not meet or exceed 50% in neutral or extended position in any horse; two horses (both operated at the site) exceeded 50% DCCR in flexion. C4-5 DDR mean values in extension did not meet or exceed 20% in any horse; one horse (operated at the site) exceeded 20% DDR in neutral position and 20 horses had mean DDR measures ≥20% in flexed position, including 11 of the 12 horses operated at this site.

Flexion statistically significantly increased DCCR C4-5 values, as compared to neutral position (median DCCR neutral 2.1%, median DCCR flexed 20.4%, Wilcoxon signed-rank p = 0.001), regardless of whether horses were operated at this site. Flexion also statistically significantly increased DDR C4-5 mean values, as compared to neutral (absolute mean difference 25.7%, paired t-test p = 0.0000), regardless of whether horses were operated at this site. DCCR in extension, as compared to neutral, was also statistically significantly increased for all horses (median DCCR neutral 2.1%, median DCCR extension 20.1%, Wilcoxon signed-rank p = 0.0001) though mean value remained below 50%.

#### Intervertebral junction C5-6.

**Fig 7:**
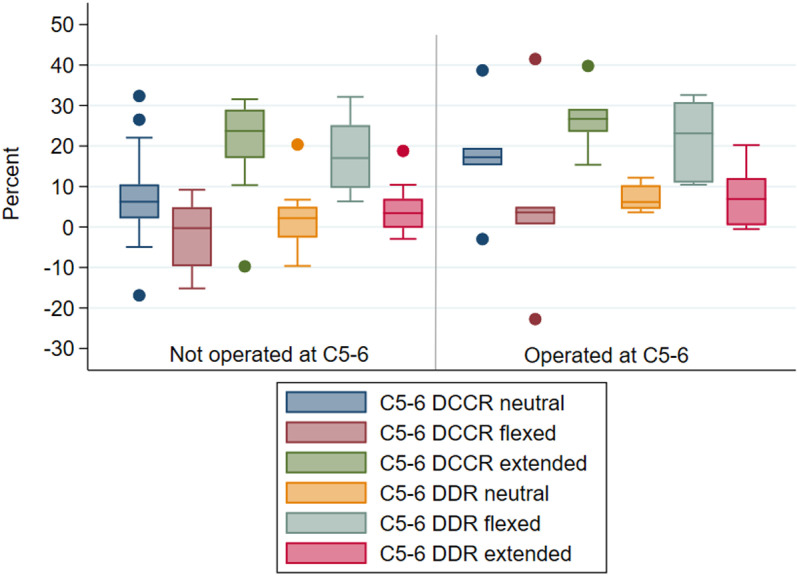
Mean observer DCCR (%) and DDR (%) at C5-6 in neutral, flexed and extended neck positions in horses that were not operated (n = 16) versus operated (n = 6) at this site. Horizontal line reflects the median.

Neutral and extended position myelographic values could be measured in 22 horses at this articulation; flexed measures were available for 21 horses (S4 Dataset). No myelographic measures (Fig 7, S4 Dataset) were statistically significantly different between horses operated at C5-6 (n = 6) and horses not operated at the site (n = 16).

No horses, including the six operated at C5-6, had mean DCCR values exceeding 50% at this articulation, regardless of positioning. Mean DDR values met or exceeded 20% in eight horses in flexed position (four operated, four not operated at this site), in one horse (not operated at this site) in neutral position, and in one horse (operated at this site) in extended position. No statistically significant associations were found between mean DDR values ≥20% and operative grouping, with no difference in DDR between horses that were or were not operated at this site (Fig 7).

As compared to neutral, for all horses, mean C5-6 DCCR values were statistically significantly decreased (wider diameter) in flexed position (median DCCR neutral 7.4%, median DCCR flexed 0.7%, Wilcoxon signed-rank p = 0.0041) and increased (narrower diameter) in extended position (median DCCR extended 25.4%, Wilcoxon signed rank p = 0.0011). In contrast, as compared to neutral, mean DDR value was statistically significantly increased (reduced dural diameter) in flexion (absolute mean difference 15.1%, paired t-test p = 0.0000) and exceeded 20%. Mean DDR value in extension was not significantly different from mean DDR value in neutral position (absolute mean difference 1.9%, paired t-test p = 0.0901).

#### Intervertebral junction C6-7.

**Fig 8:**
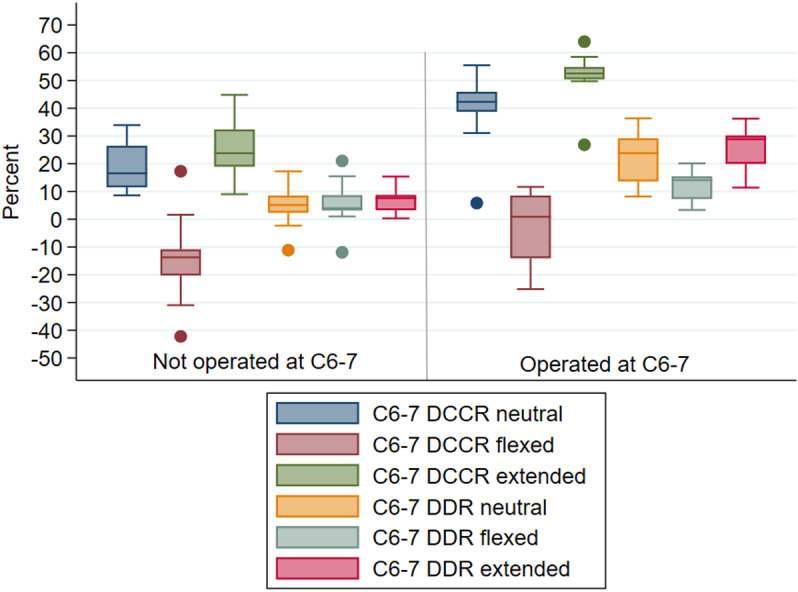
Mean observer DCCR (%) and DDR (%) at C6-7 in neutral, flexed and extended neck positions in horses that were not operated (n = 13) versus operated (n = 9) at this site. Horizontal line reflects the median.

Measurements were available for 22 horses at this articulation in neutral, flexed, and extended position (Fig 8, Table 2, S5 Dataset). Mean DCCR and DDR measures at C6-7 in neutral and extended positions were statistically significantly different after correction for multiple comparisons between the horses operated at this site (n = 9) and those not operated at the site (n = 13); and were higher (reduced diameter) in the operated horses. Neither myelographic measure in flexed position was statistically significantly different between the two groups after correction for multiple comparisons (Table 2).

**Table 2:** Mean myelographic measures at C6-7 articulation by operative group.

Measurement	Mean value horses not operated at C6-7 (95% CI)	Mean value horses operated at C6-7 (95% CI)	p value*
**DCCR neutral**	18.6% (13.4%, 23.8%)	39.3% (28.2%, 50.4%)	0.0004
**DCCR flexed**	−13.8% (−22.5%, -5.0%)	−3.3% (−13.6%, 6.9%)	0.1014
**DCCR extended**	24.3% (17.8%, 30.9%)	51.2% (43.4%, 59.1%)	0.0000
**DDR neutral**	4.9% (0.6%, 9.1%)	22.3% (15.2%, 29.5%)	0.0001
**DDR flexed**	5.9% (1.1%, 10.5%)	12.3% (7.7%, 17.0%)	0.0492
**DDR extended**	6.4% (3.9%, 8.9%)	25.4% (19.0%, 31.8%)	0.0000

*p values calculated using independent sample t-test with the exception of DDR extended which used Wilcoxon Rank-Sum due to non-normality

Two of nine C6-7 operated horses met DCCR positive criteria for compression (≥50%) in neutral position, none in flexed position, and seven of the nine in extended position (however, one of those horses was scored <50% DCCR by two observers); only DCCR ≥50% in extended position statistically significantly distinguished operated versus non-operated horses when corrected for multiple comparisons (Fisher’s exact test p = 0.000). When the single horse with discordant DCCR extended results (two observers measured <50% and one > 50%, yielding a mean measure >50%) was reclassified as “negative”, DCCR positivity (defined as ≥ 50%) in extension remained statistically significantly associated with surgery at C6-7 (Fisher’s exact test p = 0.001).

Six of the nine C6-7 operated horses met DDR positive criteria for compression (≥20% DDR) in neutral position, one of the nine in flexed position, and six of the nine in extended position. DDR ≥ 20% in neutral and extended, but not flexed, position distinguished between horses operated and not operated at C6-7 after correction for multiple comparisons (Fisher’s exact test: neutral p = 0.001, flexed p = 0.662, extended p = 0.001).

As compared to neutral, for all horses, mean C6-7 DCCR values were statistically significantly decreased in flexed position (paired t-test p = 0.0000, absolute mean difference 36.6%) and slightly increased in extended position (paired t-test p = 0.0053, absolute mean difference 8.3%). For all horses, DDR measures at this site were not statistically significantly changed by re-positioning from neutral (paired t-test neutral vs flexed p = 0.077, absolute mean difference 3.5%; neutral vs. extended p = 0.647, absolute mean difference 2.2%).

#### Correlations between DCCR and DDR measures.

At C2-3 and C3-4, DCCR and DDR measures in neutral position were poorly and non-significantly correlated (Table 3, S6 Dataset). However, in flexed position, DCCR was strongly positively correlated with DDR at C2-3. At C4-5 and C5-6, DCCR had moderate correlation with DDR in both neutral and flexed positions, but weak and non-significant correlation in extended positioning. At C6-7, DCCR and DDR measures were moderately to strongly correlated in neutral and extended positions. However, point estimates had relatively wide confidence intervals for most correlation coefficients (Table 3).

**Table 3:** Correlations of DCCR and DDR by position and intervertebral junction.

Intervertebral junction	Correlation coefficient (95% CI) DCCR neutral with DDR neutral	Correlation coefficient (95% CI) DCCR flexed with DDR flexed	Correlation coefficient (95% CI) DCCR extended with DDR extended
**C2-3**	Pearson’s r 0.251 (−0.1901, 0.608)	**Pearson’s r 0.844§** (0.623, 0.941)	not determined
**C3-4**	Pearson’s r −0.087 (−0.500, 0.358)	Pearson’s r 0.326 (−0.111, 0.657)	not determined
**C4-5**	**Pearson’s r 0.620†** (0.268, 0.826) Spearman’s rho 0.439 (0.021 to 0.726)	**Pearson’s r 0.584†** (0.215, 0.807)	Pearson’s r 0.334 (-0.114, 0.669)
**C5-6**	Pearson’s r 0.531* (0.141, 0.778)	Pearson’s r 0.508* (0.098, 0.771)	Pearson’s r 0.206 (−0.237, 0.577) Spearman’s rho -0.020 (-0.438, 0.405)
**C6-7**	**Pearson’s r 0.736**ǂ (0.456, 0.883)	**Pearson’s r 0.597†** (0.235, 0.814)	**Pearson’s r 0.798§** (0.567, 0.913) Spearman’s rho 0.797 (0.565, 0.912)

When at least one measure did not meet assumptions of normality, correlation coefficients were derived from both Pearson’s and Spearman’ methods. Bolded values achieved statistical significance.

*p < 0.05.

†p < 0.01.

ǂp < 0.001.

§ < 0.0001.

#### Interrater agreement.

Agreement of raters for sites assigned as ‘positive’ (abnormal) exceeded 80% between raters for most DCCR and DDR measures (S7 Dataset). Rater discordance was more frequent in DDR measurements (42 of 54 cases in which one rater’s measurement disagreed with the other two for meeting previously established criteria for positivity [6]). No single rater disagreed with the other two at a significantly higher frequency than others (rater 1, 19 cases discordant; rater 2, 18 cases discordant; rater 3, 17 cases discordant). Decreased DCCR measures (increased diameter) at C5-6 and C6-7 in flexion, as compared to extension, was a pattern replicated by all raters. Similarly, DDR measures increased in flexion (reduced diameter), as compared to neutral, for all raters. DDR measures at C6-7 did not exhibit a consistent pattern of change in response to position for any of the three raters.

#### Receiver operator characteristic curves.

Receiver operator characteristic curves (ROC curves) were constructed for DCCR and DDR measures [25] (S8 Dataset). Area under the curve (AUC) was <0.8 for most measures at C4-5 and C5-6, indicating poor discrimination between operative and non-operative sites. For C6-7, both DCCR and DDR in neutral and extension had good discrimination between operative and non-operative sites (DCCR neutral AUC 0.88, 95% CI 0.66, 1.00; DCCR extended AUC 0.97, 95% CI 0.89, 1.00; DDR neutral AUC 0.95, 95% CI 0.86, 1.00; DDR extended AUC 0.99, 95% CI 0.97, 1.00). Confidence intervals for AUC of these curves generally overlapped with those of ROC AUC generated from Van Biervliet’s data [6] (S8 Dataset). However, diagnostic thresholds for reduction were different between the two sets of data: for Van Biervliet’s data, C6-7 DCCR-neutral threshold ≥30% had a reported sensitivity of 100% (calculated 95% CI 29–100%) and specificity of 41% (calculated 95% CI 23–61%) for compression confirmed on histopathology [6]; the same threshold applied to DCCR-neutral in our data set yielded a sensitivity of 89% (95% CC 56–100%) and specificity of 85% (95% CI 55–98%) for prediction of operative site. Similarly, C6-7 DDR-neutral threshold ≥20% had a reported sensitivity of 100% (calculated 95% CI 29–100%) and specificity of 93% (calculated 95% CI 78–99%) for histopathologic compression; the same threshold applied to DDR-neutral in our data set resulted in a sensitivity of 66.7% (95% CI 30–93%) and specificity of 100% (95% CI 75–100%) for prediction of operative site.

#### Surgical complications.

Complications associated with cervical stabilization surgery were reported in all horses (Table 4). Non-neurologic complications documented in the medical record included pyrexia (n = 7), incisional seroma (n = 3), pneumonia (n = 2), and colic (n = 2). Two horses displayed hyporexia (n = 2). Ten horses had suspected neurologic complications. These included signs associated with Horner syndrome (n = 4, with two horses operated at C4-5 only, one at C4-5/C6-7 and one at C5-6/C6-7); severe, persistent, and unilateral thoracic pruritis lasting for months (n = 1, operated at C3-4 and C4-5); and grade 5/5 unilateral thoracic limb lameness with profound shoulder muscle atrophy (n = 1, operated C6-7). Twenty horses had upper airway endoscopy prior to surgery, with normal findings, and eight horses had repeat endoscopy performed in the week after surgery. One had developed left laryngeal hemiplegia and six had developed right laryngeal hemiplegia. Grading of paralysis was recorded in four horses with right laryngeal hemiplegia and was 4 out of 4; for two horses, the abnormalities were simply reported to be ‘mild’, and for one horse also with right laryngeal hemiplegia, as severe. Five of the horses with laryngeal hemiplegia were operated at one site only (C4-5, C5-6, and C6-7 in three horses) and two horses were operated at two sites each (C5-6 and C6-7).

**Table 4:** Surgical complications and long-term post-operative findings in 22 horses undergoing cervical interbody fusion.

Horse ID & operative sites	Post-operative complications	Select findings at neurologic re-evaluation	Ataxia grade in forelimbs at re-evaluation	Ataxia grade in pelvic limbs at re-evaluation	Select follow-up imaging findings	Final status
#4 C4-5	Grade 4 RLH & R Horner syndrome.	–	–	–	–	Improved (less stumbling) but still had deficits that made pace above walking unsafe, and horse fell again. Given away and currently not ridden.
#6 C5-6 C6-7	Grade 4 RLH & R Horner syndrome, aspiration.	–	–	–	–	Lost to follow-up.
#8 C4-5 C6-7	‘Mild’ RLH & R Horner syndrome	Pelvic limb ataxia 2y 2m post-operatively. reported by examining veterinarian 1 grade above pre-operative	Not recorded	3	External radiographs 2y 2m post-operatively: Implants NAD. Subjectively, subtle reduction of opacity and notable increase in size of C4-5 APJ but not C6-7 compared to pre-operative.	Euthanized 2y 2m post-operatively due to ongoing neurologic concerns including falling on lunge line.
#10 C5-6 C6-7	Grade 4 RLH.	At 1y post-operatively, 1 grade improved in fore- & hind.	1	2	–	Returned to pleasure riding with no major concerns.
#11 C3-4 C4-5	None recorded.	–	–	–	–	Sold 18m post-operatively, returned to jumping (2 foot 6) successfully, then used for basic riding lessons many years post-operatively. Noticeably pivots behind and is hypermetric stepping over rails.
#12 C3-4 C4-5 *Re-evaluated in study 7 y post-operatively*	Fever, diarrhea. Severe, intractable thoracic pruritis for months (knocking down fences when rubbing).	7y post operatively: 1 grade improved in fore- & 2 grades in hind; intermittent dragging of hind feet in backing.	0	0	7 years post-operatively: implants NAD. Mild progressive C3/4 and C4/5 disc space narrowing, endplate sclerosis, and smooth ventral vertebral body proliferation. Static C2/3, C3/4, C4/5, C5/6, C6/C7 articular facet osteoarthrosis, moderately reduced opacity of the C3-4 and C4-5 APJ with size increase at C3-4 (height and area) but not C4-5.	Pruritis resolved after approximately 6 months. Returned to riding and performing at expected level in dressage, and occasional non-competition jumping.
#19 C3-4 C4-5 *Re-evaluated in study 7 y post-operatively*	Mild ventral migration of implant noted in immediate post-operative radiographs.	7y post operatively: 2 grades improved in fore- & hind; moderately reduced lateral flexion of neck to both sides.	0	1	7 y post-operatively implants in same position. Mildly to moderately reduced opacity of C3-4 and C4-5 APJ relative to C5-6 with mild increase in size and area measures. C5-6 and C6-7 subjectively more sclerotic with heterogeneous remodeling and suspected foraminal stenosis.	Apparently improved only after 4 years. At 7y post-operatively is ridden occasionally and only on flat ground due to ataxia downhill. Reduced lateral flexion of neck.
#22 C5-6 C6-7	Melanoma found intra-operatively near surgical site, aborted attempt to operate C5-6.	–	–	–	–	Did not get rehabilitated or returned to work (owner elected retirement).
#23 C5-6	‘Mild’ LLH, fever, pneumonia.	–	–	–	–	Did not get rehabilitated or returned to work (owner elected retirement).
#24 C3-4 C4-5	Coughing (endoscopy not performed); returned with nasal discharge and coughing one month later (endoscopy not performed).	Radiographed at another clinic 1y 9m post-operatively after falling on lunge line and limb injury, without neurologic re-assessment.	–	–	1y 9m post-operatively: implants NAD. Minimal change in opacity of C3-4 APJ, reduced opacity of C4-5, minimal change in size of either site.	Still considered unsafe to ride 7y post-operatively and is retired.
#27 C4-5	Mild R Horner syndrome, seroma.	–	–	–	–	Euthanized over 1y post-operatively due to lack of improvement and considered unsafe to handle.
#29 C6-7 *Re-evaluated in study 8 y post-operatively*	Grade 4 RLH, severe swelling at site.	8y post-operatively, 1 grade improved in fore- & hind; hypermetric in forelimbs when head elevated, and prominent R hind hyperflexion in small circles to right.	0	1	8y post-operatively: mild peri-implant lucency, C6/C7 disc space collapse, C6-7 APJ mildly reduced in opacity but notably larger and more irregular than pre-operative images.	Continued and slowly progressing gait abnormalities after return to work. At 8y post-operatively, stumbling and tripping, problematic R hind hyperflexion, retired from riding.
#30 C4-5 C5-6	Fever and low white cell count.	2m post operatively neurologic grade unchanged.	1	2	2m post-operatively: Implants NAD.	Was performing acceptably in pleasure riding, euthanized for unrelated laminitis 2y 7m post-operatively.
#31 C4-5 C6-7	Pain, fever, hindlimb edema.	–	–	–	None performed.	Sold & returned to work at 2y post-operatively, performing satisfactorily in beginner-novice eventing and 1^st^ level dressage at 7y post-operatively.
#32 C6-7 *Re-evaluated in study 4y 8m post-operatively*	Severe R forelimb lameness followed by severe R shoulder muscle atrophy.	4y 8m post-operatively, no atrophy, stiff when circled R, pivots on R forelimb.	0	0	At 4y 8m: Implant NAD, enlarged C5-6 and C6-7 APJ, C6-7 synovial joint is narrower with more sclerotic margins. Progressive, moderate C5-6 and C6-7 degenerative joint disease. C6-7 has mild reduction in opacity relative to C5-6 but larger in height and area than pre-operative images.	Performing 2^nd^ level dressage, will often refuse to turn to the right.
#35 C4-5 *Re-evaluated in study 3y 10m post-operatively*	Pneumonia.	3y 10m post-operatively: 2 grades improved in fore- & hind.	0	0	At 3y 10m: Implant NAD. C4-5 APJ is larger, more heterogeneous and mildly reduced in opacity compared to pre-operatively.	Performing well in trail riding, occasional non-competitive jumping. Occasionally canters with different leads in front to behind.
#37 C6-7	RLH, coughing with eating, severe neutropenia and colic signs 3 days post-operatively.	None performed.	–	–	None performed.	Euthanized 11 days post operatively due to gastrointestinal rupture.
#38 C4-5 *Re-evaluated in study 3y 4m post-operatively*	Left jugular distension and delayed emptying noted at 4 & 6 months post-operatively, palpable scar tissue over left jugular.	3y 4 m post operatively, 1 grade improved in hind, remains grade 1 in fore.	1	1	At 3y 4 m: Implant NAD. Similar or mildly progressive C2-3, C4-C5 and C6-7 degenerative joint disease, with negligible changes in opacity and size.	Performing acceptably in pleasure riding; reportedly still drags front feet when backing and unsteady behind down steep slopes. Occasional tripping but quick recovery, and pivots behind on circles (R > L).
#41 C5-6	Hairline fracture at implant site 3y 5m post-operatively detected after clinical decline; horse had been back in dressage work and performing well.	External evaluation by DACVIM 3y 5m post-operatively reported comparable grades to pre-operative.	1	2	3y 5m post-operatively: radiographic myelogram unremarkable. CT myelogram: incomplete cranial C6 endplate fracture within implant. Moderate right articular process ventral axial periarticular remodeling and caudal narrowing of the right APJ with right dorsal lateral contrast attenuation and mild change in cord shape at C5-6. Hounsfield units of C5-6 APJ ~ 40% lower than C4-5.	After fracture diagnosis, facet joints C4-5, C5-6 and C6-7 injected bilaterally with methylprednisolone and shockwave performed, bisphosphonate given. Horse re-commenced light arena training 18 m later (5y post-operatively) and performing satisfactorily with occasional tripping and mild ataxia.
#43 C4-5	Transiently reduced manure production.	External evaluation by DACVIM 2y post- operatively 48 hours after falling in pasture reported comparable grades to pre-operative.	2	3	2y post-operatively: implant NAD, C5 articular facet mildly heterogeneously reduced in opacity.	Minimal improvement, still very ataxic and prone to falling 2y post-operatively, not ridden due to safety concerns.
#44 C2-3	Seroma.	–	–	–	–	Did not improve and was given away when considered unsafe to continue training and riding.
#67 C6-7	Fever, seroma.	–	–	–	–	Horse was improving by 1y post-operatively but never started in training. Retained as companion.

RLH = Right laryngeal hemiplegia.

LLH = Left laryngeal hemiplegia.

NAD = no abnormalities detected.

#### Long-term post-operative findings.

Images obtained more than 18 months post-operatively were available for 10 operated horses (Table 4), including standing cervical radiographs from 9 horses (4 operated at 2 sites, 5 operated at 1 site; total 13 operated sites) and radiographic and CT myelogram images from another horse (operated 1 site). The plain radiographs for the 9 horses were performed 4.4 ± 2.2 years postoperatively, and the myelogram images for the single horse 3.4 years post-operatively (Table 4). Six of the imaged horses had neurologic evaluation by investigators and standing cervical radiographs performed for the purposes of this study. Three horses had cervical radiographs performed during external diagnostic evaluations after the clients reported falling during non-ridden exercise. The horse that had external myelography studies performed had displayed a sudden increase in ataxia after it had been returned to training and had been considered to be performing acceptably.

**Fig 9:**
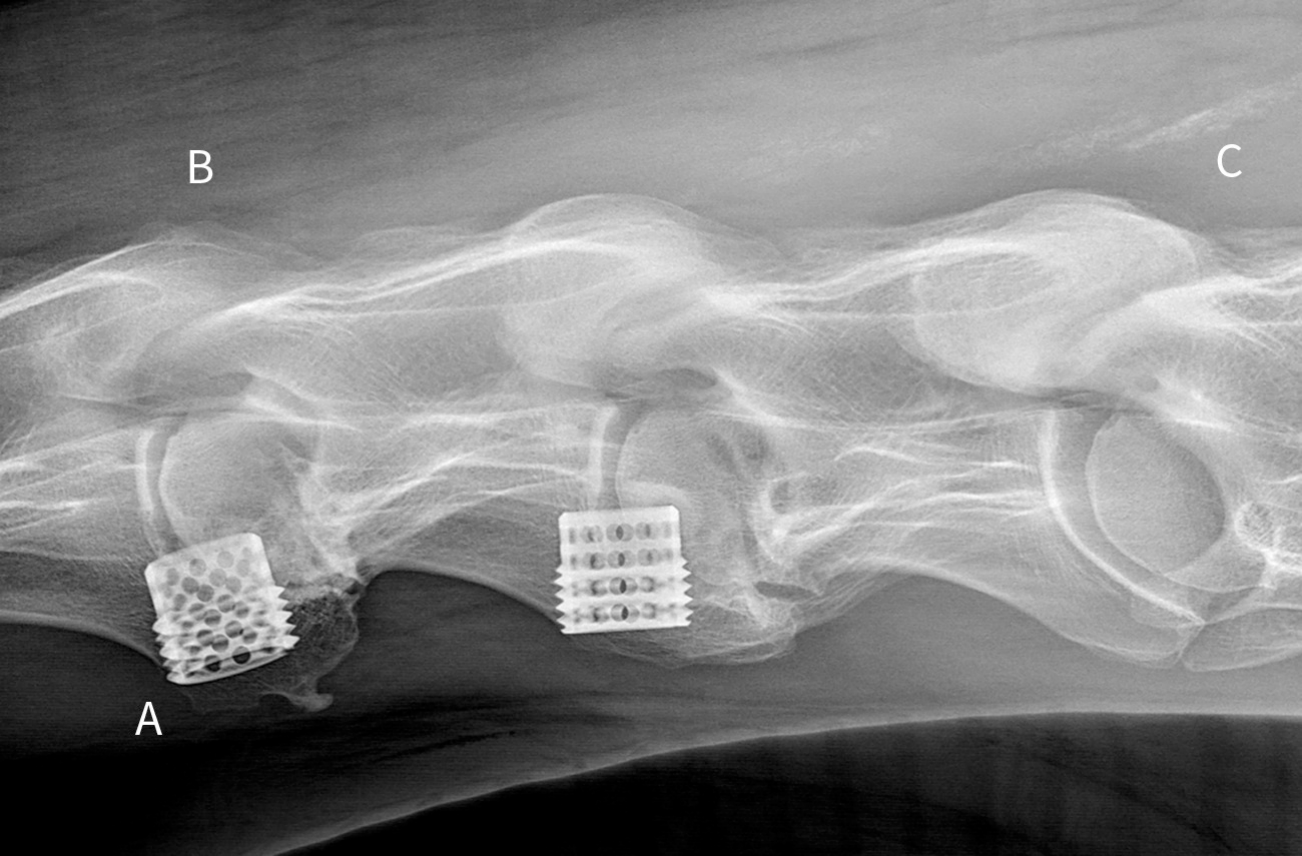
A-C: Post-operative lateral cervical radiograph acquired 7 years after cervical stabilization procedure at C3-4 and C4-5 (Horse #19). **A.** Mild ventral migration of the C3-4 cylindrical metal implant recorded soon after initial placement has remained stable over time. No implant breakage is identified. **B.** The C3-4 articular process joint is smaller and diffusely reduced in opacity, relative to the C4-5 and C5-6 articular process joints. **C.** Linear dystrophic mineralization of the dorsal cervical soft tissues.

**Fig 10:**
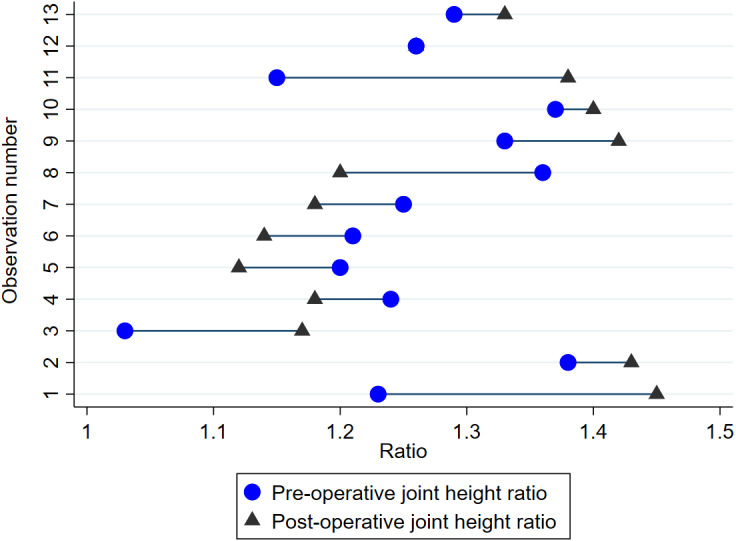
Pair plot demonstrating change in APJ joint height ratio at 13 operated articulations in nine horses imaged 4.4 ± 2.2 years postoperatively.

**Fig 11:**
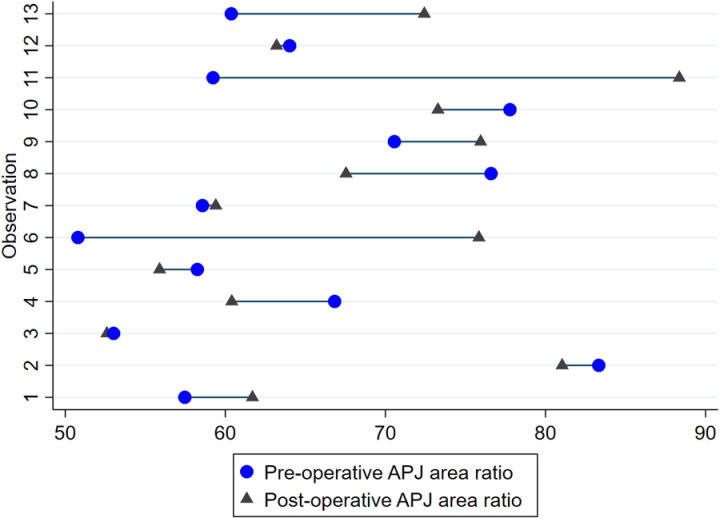
Pair plot demonstrating change in APJ joint area ratio at 13 operated articulations in nine horses imaged 4.4 ± 2.2 years postoperatively.

**Fig 12:**
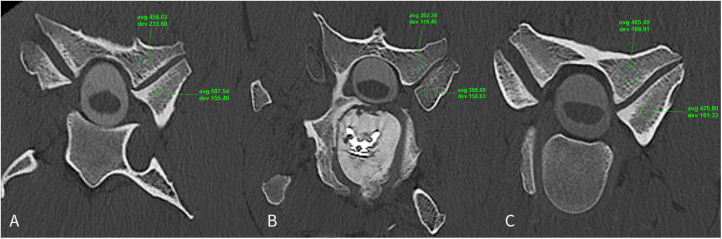
A-C: Postoperative transverse plane computed tomography images at the level of the cervical articular process joints obtained 3.4 years after cervical interbody fusion at C5-6 (Horse #41). **A.** Hounsfield measurements of the C4-5 articular process joints (cranial to the surgical site). **B.** Hounsfield measurements of the C5-6 articular process joints (at the surgical site). **C.** Hounsfield measurements of the C6-7 articular process joints (caudal to the surgical site). In images A and C, the articular processes have thick cortical and subchondral bone with well-defined, trabecular bone. In image B, the operated site, the articular processes have poorly defined bony trabecular pattern and diffusely reduced mineral attenuation and thickness of the subchondral and cortical bone.

The radiologist (LN) reported subjective mildly to moderately decreased radiographic opacity of APJ at 10 of 13 operated sites in the 9 horses, when compared to the adjacent non-operated sites in the same radiograph. The operated sites typically had a heterogeneous reduction in mineral opacity throughout the medullary cavity, with thin subchondral APJ margins, thin cortical bone, and reduced mineral opacity along the ventral margin of the articular joint (Fig 9). Mean APJ height ratio (1.25 vs 1.28, p = 0.417) and APJ area ratio (64.37 vs 68.28, p = 0.249) pre- to post-operatively for the 13 operated sites were not significantly different. The APJ height ratio increased post-operatively at 7 operated sites (suggesting an increase in APJ height), decreased at 5, and remained unchanged at 1 operated site (Fig 10). The APJ area ratio increased at 5 operated sites (suggesting an increase in APJ area), decreased at 5 and was minimally changed at 3 operated sites (Fig 11). In the single horse in which long-term post-operative myelography was performed, for radiographic myelography images it was reported that there was no abnormalities of the implant (at C5-6) and no evidence of compression at any site. On CT myelogram an incomplete cranial C6 endplate fracture within the implant was reported, with moderate right articular process ventral axial periarticular remodeling and caudal narrowing of the right APJ with right dorsal lateral contrast attenuation. The operated APJ had substantially reduced Hounsfield unit readings compared to the adjacent C4-5 and C6-7 articulations (Fig 12), indicating loss of bone density at the operated site.

For the six horses that had re-evaluation specifically for this study, two horses were not being ridden or ridden occasionally in a limited fashion (flat ground only) due to continuation of problematic gait abnormalities observed before surgery. Another horse was improved and performing in pleasure riding, reportedly with some persistent deficits reflected by tripping, pivoting behind, and unsteadiness down steep slopes (Table 4). Three horses were performing to owner satisfaction in pleasure riding, dressage and occasional non-competitive jumping, including the horse that had severe pruritis and the horse that had severe post-operative thoracic limb lameness. Pelvic limb ataxia had improved by 1 grade in four of these horses, and by 2 grades in the other 2 horses, resulting in a score of 0/5 (normal) in three horses, and grade 1/5 (mild deficits) in 3 horses with all horses graded 0 in the forelimbs on reassessment apart from one horse which was graded 1.

Long term outcome information for 21 of the 22 operated horses is provided in Table 4. For three horses, there was no attempt to rehabilitate and return to work; because one died from spontaneous gastrointestinal rupture 11 days following the cervical stabilization surgery, and two horses belonged to the same client who elected to retire them post-operatively. Eight of the remaining eighteen horses (44.4%) did not successfully return to or remain in work; two were euthanized and two were given away due to ongoing clinical signs, and four were removed from work due to ongoing neurologic deficits and retained by their owners. Another three horses (16.7%) were ridden at pleasure level, with ongoing restrictions; this includes the horse that displayed decline and minor vertebral fracture after initially successfully returning to dressage training. Seven horses (38.9%) were participating in ridden work with minimal reported concerns; four in pleasure riding, and three returned to competition work for some duration (in 2nd level dressage, beginner-novice eventing, and jumping 2 foot 6 class for each horse, respectively).

## Discussion

This study evaluated whether two currently dominant myelogram measurement methods when applied to historically obtained radiographic myelograms identified intervertebral sites previously selected for cervical stabilization in horses operated at our institution. Our expectation was that DCCR and DDR measurements would meet published criteria [6] for discrimination of operated sites versus non-operated sites. Instead, we found that neither DCCR nor DDR measurements significantly differed for horses operated at C3-4, C4-5, and C5-6 sites versus horses not operated at those sites. Two horses in the study did not have any single site that met criteria for compression in any position. Myelographic measures were different only for horses operated at C6-7, in neutral and extended neck positions, as compared to horses not operated at that site.

Interestingly, observer-measured DCCR rarely met previously published positive criteria for compression (≥50% reduction) in any horses of this surgical cohort, regardless of operative site(s). In contrast, DDR frequently exceeded the criterion for potential compression (≥20% reduction) in flexion at C3-4, C4-5, and C5-6, though neither method produced values that differed significantly between horses that were or were not operated at these respective sites. Because these contrast column reduction criteria did not accurately classify operated from non-operated horses, we evaluated contrast column reduction thresholds using receiver-operator characteristic curve (ROC) analyses. Discriminative ability was poor for most measurements at C4-5 and C5-6 (ROC AUC < 0.8). Discriminative ability was good to excellent for DCCR and DDR in neutral and extended positions at C6-7 (ROC AUC > 0.85). However, cutpoint estimates from our ROC analyses for C6-7 differed to those of previously published criteria [6]. Discordant thresholds are partially explained by the small number of horses in this and previous studies, which result in wide confidence intervals (CI) for diagnostic accuracy indices. The confidence intervals for sensitivity (Se) and specificity (Sp) reported here substantially overlap with those from a criteria-setting publication for these specific methods [6].

In the current study, positioning had a strong impact on contrast column reduction that varied by intervertebral site. At C3-4 and C4-5, DCCR and DDR both increased (became narrower) in flexed position as compared to neutral. However, at C5-6 and C6-7, DCCR decreased (became wider) in flexion as compared to neutral, accompanied by unexpected divergence of DCCR and DDR at the C5-6 site where DDR narrowed in flexion. Our results support that spinal canal dimension at the C6-7 articulation is also prone to dynamic changes; for horses operated at this site, DCCR and DDR values were significantly altered by flexed or extended positioning, with flexion providing a decompressing effect. Clearly, positioning of the neck is important in detecting dynamic changes in canal diameter, potentially more so when disease is present. Similarly, an MRI study of human cervical stenosis demonstrated a substantial risk of failure to identify stenotic lesions if dynamic positioning was neglected [26]. It should also be considered that different operators may apply different degrees of flexion and extension to horses’ necks during myelogram procedures, which potentially could also influence conclusions from the resulting images.

We also report generally poor correlation between concurrently measured DCCR and DDR values at the same articulation. Our hypothesis was that dural diameter and dorsal myelographic column reductions are both proxy measures for intervertebral spinal canal dimension and the two measures would correlate and agree for diagnostic classification based on previously published criteria. In fact, correlations were not robust for most measures at most sites. We conclude that DCCR and DDR measures do not appear to be interchangeable and may produce different decision outcomes at articulations within the same individual.

We considered the possibility that interrater variation could account for our results; however, interrater reliability for diagnostic classification using DCCR ≥50% and DDR ≥ 20% criteria exceeded 80% for most measurements. Also, no single rater was more likely to be discordant with the other two raters. Interrater variation does not appear to explain our results.

Recently, computed tomography has been promoted in evaluation of equine spinal ataxia cases, since enhanced imaging of bone and soft tissues allows sensitive detection of a range of lesions, in addition to detection of lateralized compression [27]. Scanning the entire equine cervical spine requires specific equipment to permit evaluation beyond the three most cranial cervical articulations, and there may still be limits to the ability to manipulate head and neck position [28]. In the current study, flexion of the head, as compared to neutral, changed both DCCR and DDR values substantially at most articulations: DCCR was significantly increased (narrower) in flexion at C3-4 and C4-5, decreased (wider) in flexion at C5-6 and C6-7, and increased in extension at C5-6. DDR was significantly increased in flexion versus neutral at C2-3, C3-4, C4-5, and C5-6. It is therefore possible that some dynamic compressive lesions may be challenging to evaluate by CT if broad ranges of head and neck position cannot be accommodated by the equipment used. Furthermore, detection of other abnormalities, such as the bony fragment identified in one of the cases in the current study, or false compression induced by positioning may attract surgical attention towards a specific site, reducing attention to other potentially abnormal locations. The advent of dynamic computed tomography imaging for the equine neck will likely permit the clearest assessment of interactions between bony structures and soft tissues of the equine neck and spinal cord [29].

There were a high number of neurological complications observed in operated horses in this study, including the development of laryngeal hemiplegia in seven of eight horses that underwent airway examination post-operatively, and which was functionally severe in five. It was unclear whether these horses were specifically selected for reassessment based on specific post-operative clinical signs such as coughing; nevertheless they represent nearly one third of our surgical cohort which is a much higher proportion than previously reported [2,11,17]. This suggests that pre- and post-operative upper airway endoscopy is an essential adjunct to surgical cervical stabilization, and that clients should be informed of the risk of laryngeal hemiplegia in case it has implications for the horse’s athletic future. All surgeries in this study were performed by a single experienced surgeon, suggesting that specific features of surgical approach or equipment utilized might explain the high incidence, rather than experience. Other significant neurologic complications were also observed, and were in some cases profound. The onset of severe prolonged unilateral pruritis in one horse operated at C3-4 and C4-5, and of severe lameness and muscle atrophy in a horse operated at C6-7 were suspected to relate to nerve impingement. These signs eventually resolved after many months and both horses reportedly returned to an expected level of performance. There appeared to be no clear association of neurologic complications with operation of a single site versus two sites, or with specific sites that were operated.

Comparison of radiographic changes between pre- and post-operative images obtained years apart is challenging because of factors including patient differences in proximity to the imaging plate, differences in degree of obliquity, and variability in radiographic equipment quality and settings. However, when operated sites were compared with adjacent non-operated sites in the same postoperative image, consistent, mild reduction in radiographic opacity of APJ was identified at most of the evaluated operated sites. However, there was no definitive evidence of reduction in APJ size, as measured by height or area at the operated sites many years later, and adjacent non-operated sites did not have a consistent change in opacity or size. APJ thickness and changes to the spinal canal lumen could not be objectively assessed in this study. Other features that might result in clinical improvement in operated horses could include atrophy of regional soft tissues, stabilization of dynamic motion, and prevention of progressive arthritic changes. Repeat CT myelography would provide the most comprehensive assessment of post-operative changes in operated horses, but is not routinely performed or commonly available in clinical practice. Cylinder implants were apparently stable in reassessed horses, and pathologic fractures were not identified with the exception of the horse that underwent CT evaluation over three years post-operatively due to unexpected escalation of neurologic deficits. In this horse the fracture line was not visible radiographically. There was evidence of substantial reduction in bone density at the operated site of this horse based on Hounsfield unit measurements, suggesting that osteopenia potentially contributed to this complication.

Four of the six horses that were specifically re-evaluated for this study had returned to and remained in ridden work, including one with remaining deficits. Five of the re-evaluated horses had at least one site with a DDR value of at least 25% that was not operated, and three of them a site with DDR ≥30% that was not operated. This might suggest that current DDR values to designate compression are conservative, and higher values more appropriate for reflecting pathologic cord compression; that surgical intervention at other sites was adequate in these horses to confer stability or improvement in non-operated affected sites; and/or that other features associated with surgery including pharmacologic therapies, rest, rehabilitation, and carefully regimented return to exercise were responsible for perceived improvements in these horses. Surgeons may not operate all potentially affected sites in an individual because of reasons including owner concerns or desires, intra-operative complications, or multiple sites of compression. Also, surgeons may potentially operate sites that do not meet compressive criteria on radiographic myelogram to prevent progression of arthritic changes or to stabilize dynamic or misaligned sites, or may select sites with abnormal sagittal ratio measurements.

In the current study, approximately 55% of all operated horses that underwent rehabilitation attempts either did not return to ridden exercise, or had gait concerns that ended or complicated their use. The reasons for these suboptimal outcomes in a group without competitive athletic emphasis are unclear, though our findings are comparable with a recent study reporting 43% return to use in surgically treated horses, in a population similar to the current study (warmblood dominant, mostly mature horses, pleasure riding emphasis) [17]. Though neuromuscular disorders other than spinal cord compression could have caused clinical signs in some of our subjects, nearly all horses had negative equine protozoal encephalomyelitis testing and normal cerebrospinal cytology results. Equine degenerative myeloencepthalopathy, however, is more challenging to detect and exclude, and has clinical similarities to cervical cord compression. The possible contributions of surgically unaddressed, compressed sites, orthopedic disease, or minor vertebral fractures as noted in one case in this series must also be considered.

This study was limited by a relatively small number of horses, as well as the lack of a diagnostic gold standard for cord compression in the form of spinal cord histopathology. Surgical site selection was ultimately determined by collaboration between several individuals, using heavy reliance on DCCR evaluation which was the most frequent assessment in many institutions at the time these cases presented. Diagnostic utility of different myelography measurements remains a source of debate [9]. However, the findings of the current study indicate that there can be discordance between different diagnostic systems, as well discordance between DCCR and DDR measurements. These findings have implications for selection of surgical sites. These issues are further complicated by inter-observer agreement challenges and marked dynamic changes in myelographic measures with head positioning, as well as the limited capacity for radiographic myelography to detect lateralizing lesions. Given the risks and costs of stabilization surgery, additional studies are needed to develop more robust criteria for determining spinal cord compression on radiographic myelograms and to further assess the utility and outcomes of surgical stabilization versus conservative therapies in affected horses. In conclusion, the findings of this study indicate that multiple pieces of information, including the results of detailed neurological examinations, additional comprehensive diagnostic testing, client expectations and risks must be evaluated when planning a therapeutic strategy for horses with spinal ataxia.

## Supporting information

S1 Dataset Site C2-3 Summary Values.
(XLSX)

S2 Dataset Site C3-4 Summary Values.
(XLSX)

S3 Dataset Site C4-5 Summary Values.
(XLSX)

S4 Dataset Site C5-6 Summary Values.
(XLSX)

S5 Dataset Site C6-7 Summary Values.
(XLSX)

S6 Dataset Correlations between DCCR and DDR.
(XLSX)

S7 Dataset Interrater agreement data.
(XLSX)

S8 Dataset Receiver Operator Characteristic Curves.
(XLSX)
